# Raman‐based label‐free microscopic analysis of the pancreas in living zebrafish larvae

**DOI:** 10.1002/2211-5463.70163

**Published:** 2025-11-25

**Authors:** Noura Faraj, Eline M. F. de Lange, Klaas A. Sjollema, Ben N. G. Giepmans

**Affiliations:** ^1^ Department of Biomedical Sciences University Medical Center Groningen, University of Groningen The Netherlands

**Keywords:** E‐CARS, F‐SRS, live‐cell imaging, living zebrafish, pancreas

## Abstract

Advanced microscopy techniques, combined with a diverse set of fluorescent probes, provide valuable tools for uncovering insights into biological systems and addressing fundamental research questions. However, the need to develop and use genetic tags and probe markers presents notable challenges. Coherent Raman scattering microscopy offers a label‐free alternative, enabling live‐cell imaging of cellular structures without the need for labeling. Leveraging the benefits of Raman microscopy, we aim to analyze the pancreas in living zebrafish larvae and to evaluate chemical changes in pancreatic exocrine and endocrine compartments following exocrine damage. Here, we present a protocol for Raman‐based label‐free microscopic analysis of the pancreas in living zebrafish larvae. Using forward stimulated Raman scattering (F‐SRS) and epi coherent anti‐Stokes Raman scattering (E‐CARS), zebrafish pancreatic structures were analyzed and validated. Vibrational Raman spectra between 450 and 3100 cm^−1^ were acquired to identify chemical structural features within pancreatic regions. Raman imaging allows discrimination of distinct structures at 2850 and 2934 cm^−1^ in pancreatic exocrine and endocrine regions, which could mainly correspond to lipids and proteins, respectively. Exocrine damage causes a significant reduction in both the number and size of exocrine granules. Moreover, changes at 2934 cm^−1^ suggested chemical alterations in both exocrine and beta‐cell regions. In conclusion, SRS and CARS provide a powerful, label‐free approach for live‐cell imaging and chemical analysis in islet biology. Given the relative straightforward applicability in the pancreas, we anticipate broad implementation of Raman microscopy in other organs and across various biomedical research fields.

## Graphical abstract

Forward stimulated Raman scattering (F‐SRS) and epi coherent anti‐Stokes Raman scattering (E‐CARS) allow label‐free discrimination of distinct subcellular structures in the pancreas of living zebrafish larvae. Given the straightforward applicability, we anticipate broad implementation of Raman microscopy in other organs and across various biomedical research fields.

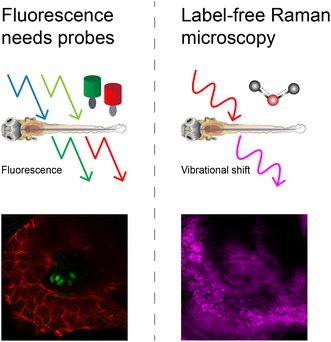

**Abbreviations**
DIC: differential interference contrast
dpf: days post fertilization
E‐CARS: Epi coherent anti‐stokes Raman scattering spectroscopy
F‐SRS: forward stimulated Raman scattering
NFP: nifurpirinol
NTR: nitroreductase
SHG: second‐harmonic generation

## Introduction

Light microscopy, paired with a diverse set of fluorescent probes, has significantly advanced the understanding of *in vivo* biological processes, cellular behavior, and protein dynamics. Genetically encoded markers such as green fluorescent protein (GFP) have enabled live subcellular imaging of proteins of interest as well as cellular processes. Fluorescent probes‐based imaging provides a powerful alternative to traditional techniques that rely on antibodies for imaging fixed samples [1, 2, 3]. Furthermore, phase contrast and differential interference contrast (DIC) microscopy allow label‐free imaging with enhanced contrast in unstained, live samples. Phase contrast is particularly effective for observing transparent or thin specimens, while DIC provides enhanced edge contrast, making the technique valuable for detailed imaging of cellular structures [4]. However, live‐cell fluorescence microscopy requires exogenous probes or genetic modifications and suffers from phototoxicity and photobleaching [5, 6], while phase contrast and DIC are limited to studying thin samples.

Advanced label‐free imaging technologies are emerging as powerful tools for uncovering novel targets. For instance, second‐harmonic generation (SHG) where two photons interact with a material, producing a new photon with twice the energy of the originals [7]. Another key technique is Raman scattering, first discovered in 1928 [8], which arises from the inelastic scattering of light due to interactions with molecular vibrations—features that are highly sensitive to changes in chemical composition and molecular structure. Therefore, Raman spectroscopy can detect subtle molecular differences and provide insights into morphological and biochemical changes within biological samples. However, spontaneous Raman scattering is inherently weak and inefficient when using a single light source [9, 10], limiting its routine application in live‐cell imaging for label‐free analysis of cellular and subcellular structures. Although label‐free data can uncover unknown changes in chemical composition, it often still requires the specificity of the fluorescent toolbox [2] to properly interpret the data. Therefore, it remains beneficial to combine label‐free with fluorescent imaging, in order to highlight the synergistic and complementary nature of this technique.

To overcome the limitations of Raman spectroscopy, advanced Raman microscopy techniques such as coherent anti‐Stokes Raman scattering (CARS) and stimulated Raman scattering (SRS) have been developed. SRS and CARS imaging offer enhanced signal strength and imaging speed by utilizing two laser beams to efficiently excite molecular vibrations. This Raman‐based imaging enables the identification and localization of different chemical species. In SRS microscopy, two laser beams (pump and Stokes) are focused on a specific region of the sample. When the frequency difference between the two lasers aligns with the vibrational resonance of a particular molecular bond, the corresponding molecules begin to vibrate, producing scattered signals that can be detected. Furthermore, CARS microscopy involves the absorption of additional photons, resulting in the emission of blue‐shifted photons known as the anti‐Stokes beam [11, 12]. Compared with fluorescence labeling and advanced microscopy techniques, Raman provides distinct advantages in living systems, including low phototoxicity and no photobleaching. Additionally, Raman microscopy offers broad label‐free applications across a range of biomedical fields and species [13, 14]. In our research, the pancreas is an organ of interest due to its central role in metabolic regulation and diseases, such as type 1 diabetes. To monitor dynamic biological processes and investigate disease mechanisms, such as islet biology and diabetes, zebrafish larvae serve as a valuable *in vivo* model [15]. Their small size, transparency, and structural similarities to the human pancreas make them particularly well‐suited for advanced microscopy techniques and islet‐exocrine interaction studies. Notably, the larval zebrafish pancreas [5 days postfertilization (dpf)] resembles the human pancreas, with the endocrine islet of Langerhans surrounded by exocrine tissue [16, 17]. Our previous work demonstrated that exocrine tissue damage can trigger beta‐cell stress and loss in zebrafish larvae [18]. To eliminate the reliance on transgenic lines, we now assess the potential of Raman‐based imaging as a label‐free approach to analyze both pancreatic exocrine cells and endocrine cells in living zebrafish larvae under untreated and treated conditions.

## Materials

1. Zebrafish lines and treatment reagents
  - Wild‐type (WT) AB zebrafish (*Danio rerio*)
  - Double transgenic *ela3l : myrpalmdevd‐mScarlet‐ntr;cryaa : venus;insulin : gfp* zebrafish, in short Tg(*ela3l :ntr;insulin : gfp*), resulting in NTR‐mScarlet‐positive exocrine cell membranes and GFP‐positive beta cells. The nifurpirinol (NFP)–nitroreductase (NTR) system induces disruption of exocrine cells as detailed recently [18].
  - 60× E3 medium (283 mg NaCl,13.3 mg KCl, 2.9 g CaCl2·2H2O, and 81.7 mg MgCl2·6H2O per liter), diluted to 1× before use
  - 200 μm 1‐phenyl 2‐thiourea (PTU) (103‐85‐5; Sigma‐Aldrich, Amsterdam, Netherlands)
  - 200 mg·L^−1^ Ethyl 3‐aminobenzoatemethanesulphonate (MS‐222/Tricaine) (886‐86‐2; Sigma‐Aldrich)
  - 5 μm NFP (20022446; LGC Standards GmbH, Lancashire, UK) in 0.1% DMSO
  - 0.1% DMSO (15 493‐8; Sigma‐Aldrich)
2. Sample preparation and SRS, CARS, SHG, and two photon microscopy
  - 10× phosphate‐buffered saline (PBS; 2.3 g NaH2PO4, 14.4 g Na2HPO4, 80 g NaCl, 2 g KCl per liter H2O), diluted to 1× before use
  - 4% paraformaldehyde (PFA) (from Formaldehyde solution 37%, Sigma‐Aldrich, 1.4003.1000; diluted in 1× PBS)
  - 1% low melting agarose in PBS or 1× E3 medium (9012‐36‐6; Thermofisher, Groningen, Netherlands)
  - Glass bottom, round imaging dish (35 mm; Merck life Sciences, Amsterdam, Netherlands)
  - STELLARIS 8 coherent Raman scattering confocal microscope (Leica Microsystems, Mannheim, Germany) containing a 20×/0.75 or 40×/1.10 HC PL IRAPO water immersion objective, a S1 oil condenser in a water dipping configuration and a CARS 2000S BP Filter cube (article no.: 158002711), with integrated laser system (picoEmerald™ S; Applied Physics & Electronics, Inc., Berlin, Germany)
3. Image analysis
  - Fiji software (imagej version 2.14.0) [19] was used to visualize the data. All images were saved as 8‐bit TIF files. The black‐and‐white images were artificially assigned a color (red, green, or blue) and overlayed in the merged channel. The mean intensity of the F‐SRS, E‐CARS and GFP signals from 3100 to 450 cm^−1^ was plotted in Fiji using Image ➔ Stacks ➔ Plot *Z*‐axis Profile. The scale bars were added via Analyze ➔ Tools ➔ Scale Bar. No segmentation or code was used to analyze the data.
  - GraphPad Prism (graphpad Software version 10.2.3, Boston, MA USA; www.graphpad.com) was used to create the violin or scatter plots.

## Methods

### Sample preparation and imaging of the pancreas in zebrafish larvae

To analyze the pancreatic islet and exocrine tissue in zebrafish, conventional fluorescent imaging of transgenic zebrafish larvae containing fluorescent proteins was initially used for detailed assessment and validation, followed by label‐free Raman imaging for further analysis.

**Fig. 1:**
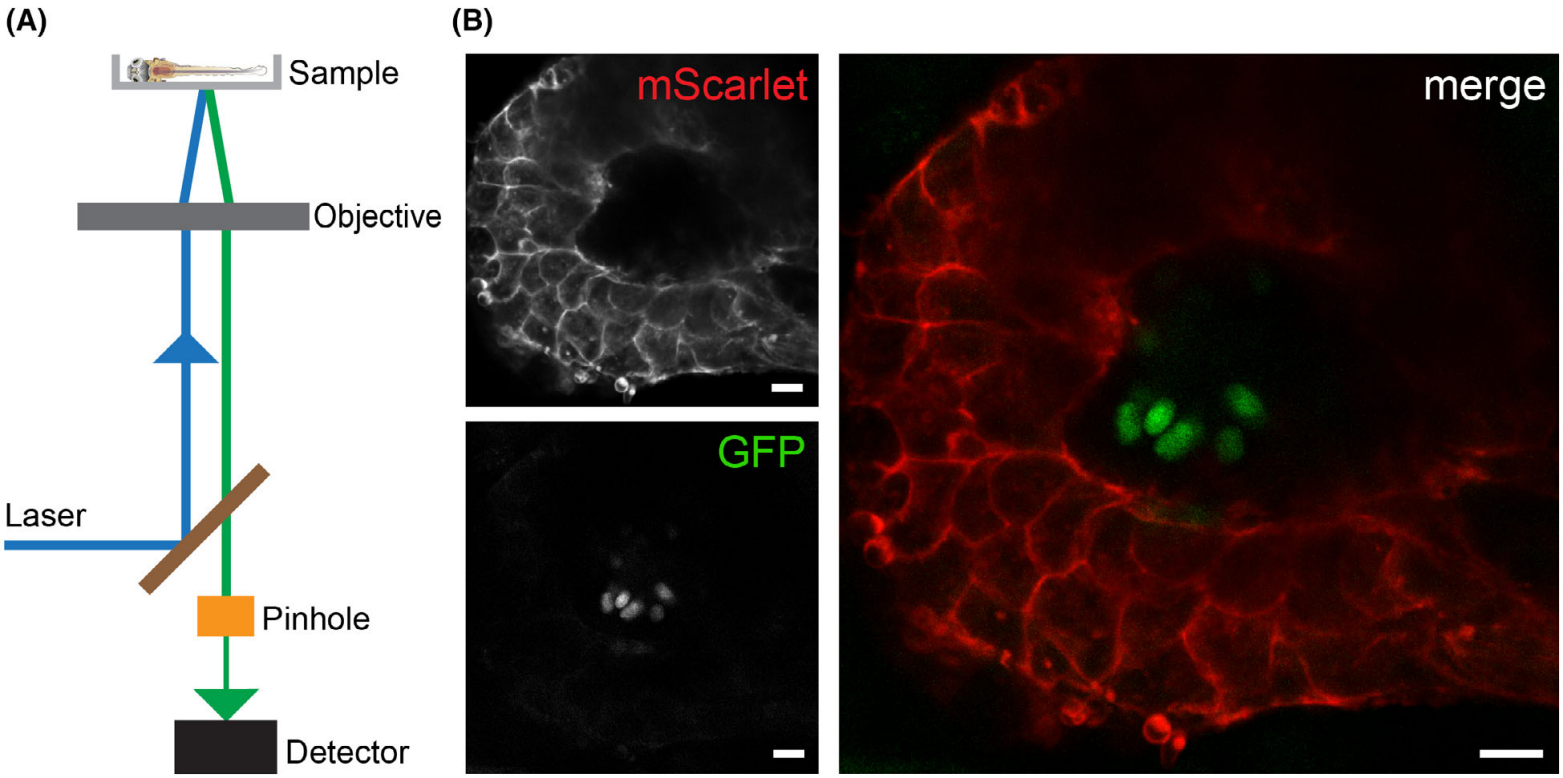
Genetically encoded fluorescent proteins allow analysis of transgenic zebrafish. To introduce the current highly used fluorescent microscope and the morphology of the zebrafish pancreas, fluorescently labeled proteins of interest are shown. (A) Optical layout for fluorescent microscopy. The sample is excited using a laser, directed through the objective to the sample and back via a pinhole before reaching the detector. (B) Fluorescent images of the exocrine region of the pancreas (mScarlet, red) and beta cells (GFP, green) together with the merged channel, in fixed, Tg(*ela3l : myrpalmdevd‐mscarlet‐ntr;cryaa : venus;insulin : gfp*), in short, Tg(*ela3l : ntr;insulin : gfp*) zebrafish larvae. Scale bars: 10 μm. GFP, green fluorescent protein.

**Fig. 2:**
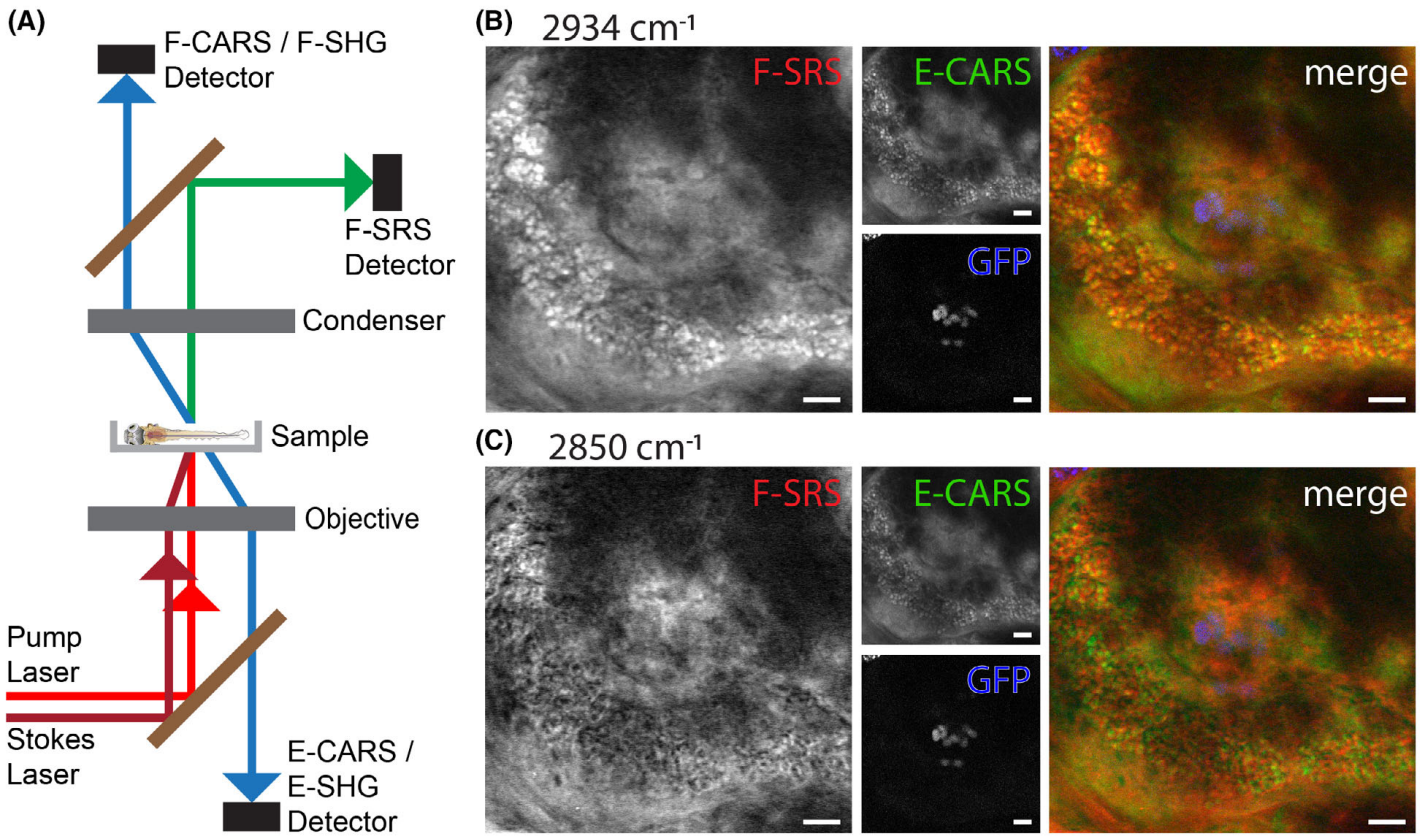
Label‐free Raman imaging in zebrafish. As an introduction to label‐free microscopy compared with conventional fluorescent microscopy, a schematic of the light path of the Raman microscope is shown. Next to this, example images of the same zebrafish pancreas as in Fig. 1 are visualized, highlighting different cellular components also detected using fluorescent microscopy. (A) Microscope setup for Raman imaging. A fixed Stokes laser and a tunable picosecond pump laser are used for SRS, CARS, and SHG microscopy. The vibrational bonds in the sample are detected via transmitted light in the forward direction (F‐SRS, F‐CARS, and F‐SHG) or reflected in epi‐direction only for CARS and SHG (E‐CARS and E‐SHG). The fluorescent (GFP) signal is detected using the forward (F‐SHG) or epi/backwards (E‐SHG) detector. (B) Raman signal at 2934 cm^−1^ in a fixed zebrafish is detected using F‐SRS (red), E‐CARS (green), and the fluorescent signal is visualized using the E‐SHG detector (GFP, blue). (C) Raman signal of the same region as in (B), now at 2850 cm^−1^. The same fish and region are depicted as in Fig. 1B. Note the use of arbitrary colors for clarity in the merged channel. Scale bars: 10 μm. SRS, stimulated Raman scattering; GFP, green fluorescent protein.

1. House AB (WT) or Tg(ela3l : ntr;insulin : gfp) zebrafish at 28 °C and maintain in 1× E3 medium
2. Add 200 μm PTU, and remove nonviable and malformed embryos after 1 dpf
3. Treat only double Tg(ela3l : ntr;insulin : gfp) larvae with 5 μm NFP in 0.1% DMSO, or only 0.1% DMSO as a control, for 12 h.
4. Euthanize larvae using 4000 mg·L^−1^ MS‐222/Tricaine
5. Fix larvae with 4% PFA at 4 °C overnight
6. Wash larvae with 1× PBS
7. Embed the larvae laterally in 1% low melting agarose in 1× PBS, in glass bottom imaging dishes, as the pancreas is located on the right side [20]. Top up with 1× PBS.
8. Image 5 dpf AB or double transgenic larvae with the STELLARIS 8 (or other) Raman microscope, using excitation of 488 nm for GFP and 561 nm for mScarlet and detected at 494–572 nm and 593–750 nm, respectively.
9. Fluorescence imaging results in NTR‐mScarlet‐positive exocrine cell membranes and GFP‐positive beta cells at 5 dpf (Fig. 1A,B).
10. Image the same larvae using Raman microscopy at 2850 and 2934 cm^−1^ using a CARS 2000S BP Filter cube (article no.: 158002711). An integrated laser system was used to produce two synchronized laser beams. Acquire data using a 300‐mW Stokes beam (1031.1 nm) and a 150‐mW tunable pump beam (720–980 nm).
11. Detect the SRS signal in the forward detection, and the CARS signals both in forward and in backward direction (F‐ and E‐CARS, respectively). The SHG signal is detected in backward (epi) direction. Acquire images with a 1024 × 1024 pixel frame size and averaging two times. Acquisition times for the images were consistently fast (Table 1).
12. Zebrafish larvae display the primary islet surrounded by exocrine granules (Fig. 2A,B).

Thus, the pancreas, which can be displayed in transgenic zebrafish larvae with fluorescence labeling, can also be analyzed label‐free using Raman microscopy, with an acquisition time comparable to that of fluorescence imaging.

### Pancreatic characteristic features are discriminated in the zebrafish larvae

Identifying distinct chemical and structural features within the pancreas, including the islet region, could provide valuable insights into its chemical composition and organizational structure.

**Fig. 3:**
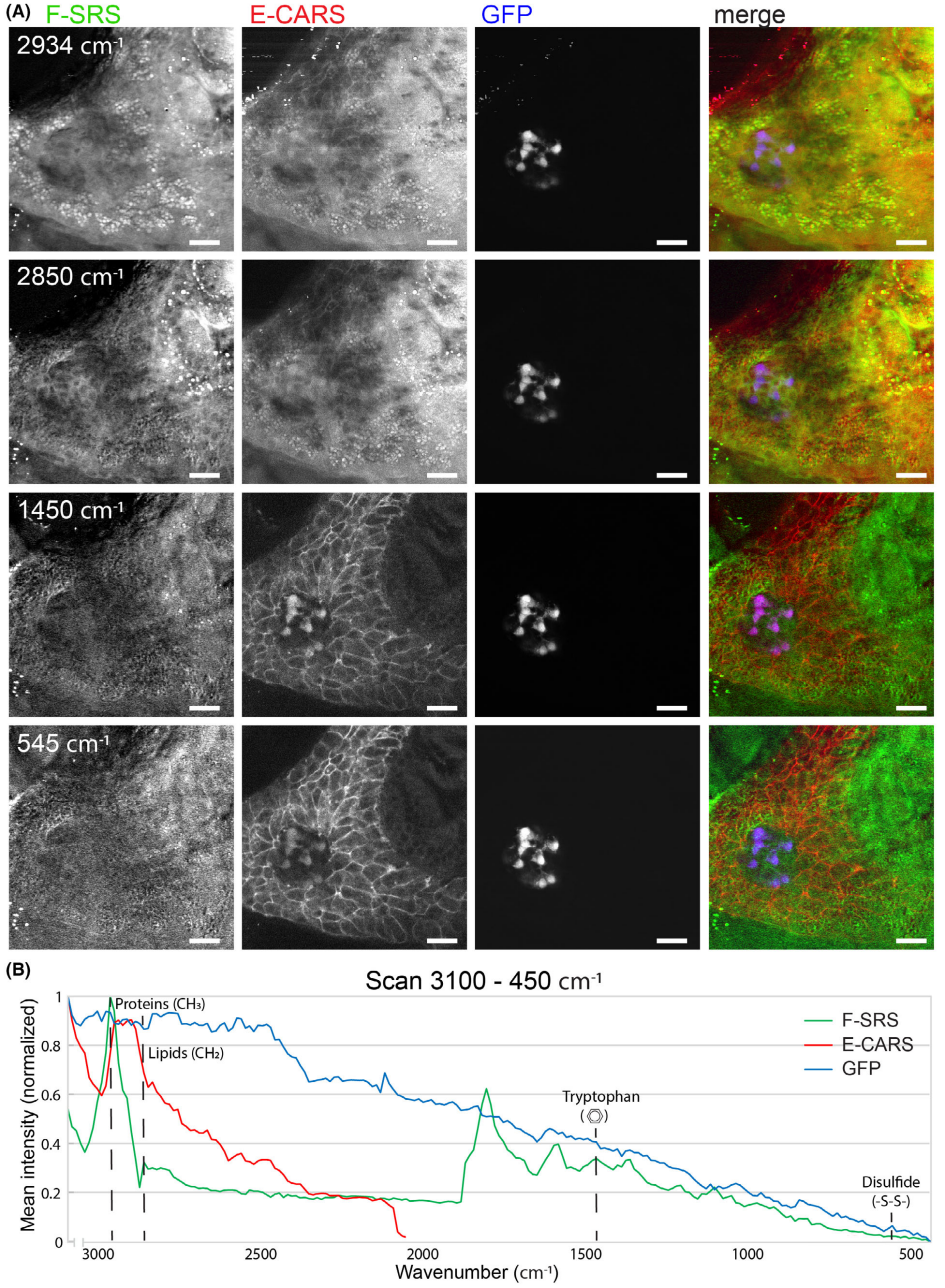
Specific Raman wavenumbers highlight different features in the pancreas. To address whether Raman is able to discriminate between different cells and granules, a scan of ascending wavelengths (depicted in cm^−1^) is performed, highlighting various features at specific wavelengths. (A) Selected wavenumbers based on a Raman sweep of 3100–450 cm^−1^ reveal different features in the pancreas (see Movie S1). The F‐SRS signal shows clear exocrine granules at 2934 cm^−1^ (top, green). The individual endocrine cells are more clearly highlighted using 2850 cm^−1^ (top middle). Lower wavenumbers (fingerprint region) show similar SRS results at 1450 and 545 cm^−1^. E‐CARS signals are only visible in the high wavenumber region here (2000 cm^−1^) and are comparable. In the lower regions, the fluorescent signals become apparent. The GFP signal, measured using the E‐SHG detector, shows the beta cells in all cases. (B) Scan of the F‐SRS, E‐CARS, and GFP signals from 3100 to 450 cm^−1^ shows the mean intensity of the total pancreatic area in (A). The vertical dashed lines correspond to protein‐rich CH_3_ and lipid‐rich CH_2_ regions detected at 2934 and 2850 cm^−1^ respectively, using SRS. Tryptophan and disulfide bonds (insulin) are detected at 1450 and 545 cm^−1^. E‐CARS is only detected and visualized at high wavenumbers up to 2000 cm^−1^, due to the selected filter set. Scale bars: 10 μm. SRS, stimulated Raman scattering; GFP, green fluorescent protein.

13 Repeat Steps 1–8
14 Image the larvae using a Raman microscope with a wide range of vibrational frequencies (450–3100 cm^−1^), using forward SRS (F‐SRS) and epi‐CARS (E‐CARS). The pump laser was tuned from 781.35 to 985.3 nm (3100–450 cm^−1^) and later specifically to 791.6, 796.9, 897.0, and 976.2 nm, respectively, to excite a vibration at 2937, 2850, 1450, and 545 cm^−1^.
15 Raman images can be sequentially captured and analyzed to uncover specific pancreatic features (Fig. 3A, Movie S1a,b and Fig. S3A). The spectroscopic characters, as reported in previous studies [21, 22, 23], were examined in the zebrafish pancreas, including CH_3_ methyl groups (2934 cm^−1^), CH_2_ symmetric stretching (2850 cm^−1^), aromatic rings (1450 cm^−1^), and disulfide bonds (545 cm^−1^). The GFP signal gradually diminishes over the imaging period due to photobleaching (Fig. 3B). In WT and transgenic larvae, both F‐SRS and E‐CARS imaging display the principal islet and exocrine granules at 2934 and 2850 cm^−1^, with the pancreatic features becoming less distinct in the SRS channel at 1450 and 545 cm^−1^.
16 CARS signals are only visible at high wavenumbers (3100–2000 cm^−1^). Fluorescence signals typically co‐appear and become more apparent at lower wavenumbers in E‐CARS microscopy [24]. Therefore, only fluorescence signals are detected in the E‐CARS channel at 1450 m^−1^ and 545 cm^−1^ wavenumbers, an observation that will be addressed in the discussion section (Fig. 3B), while no similar observations in pancreas of WT zebrafish larvae (Fig. S3A).
17 The defined pancreatic features are further supported by the presence of similarly structured GFP‐positive beta cells and mScarlet‐positive exocrine cells (Fig. S1).

Thus, 2934 and 2850 cm^−1^ are the optimal wavenumbers for label‐free observation of zebrafish pancreatic islets embedded in exocrine tissue. Analysis of the pancreas at the selected wavenumbers provides a valuable platform for examining any transformations in the chemical composition.

### Live‐cell imaging of label‐free pancreas in living zebrafish

To investigate the applicability of Raman in living systems, live‐cell imaging was conducted in 5 dpf AB or Tg(*ela3l : ntr;insulin : gfp*) larvae, following the F‐SRS and E‐CARS spectral scanning of the pancreas.

**Fig. 4:**
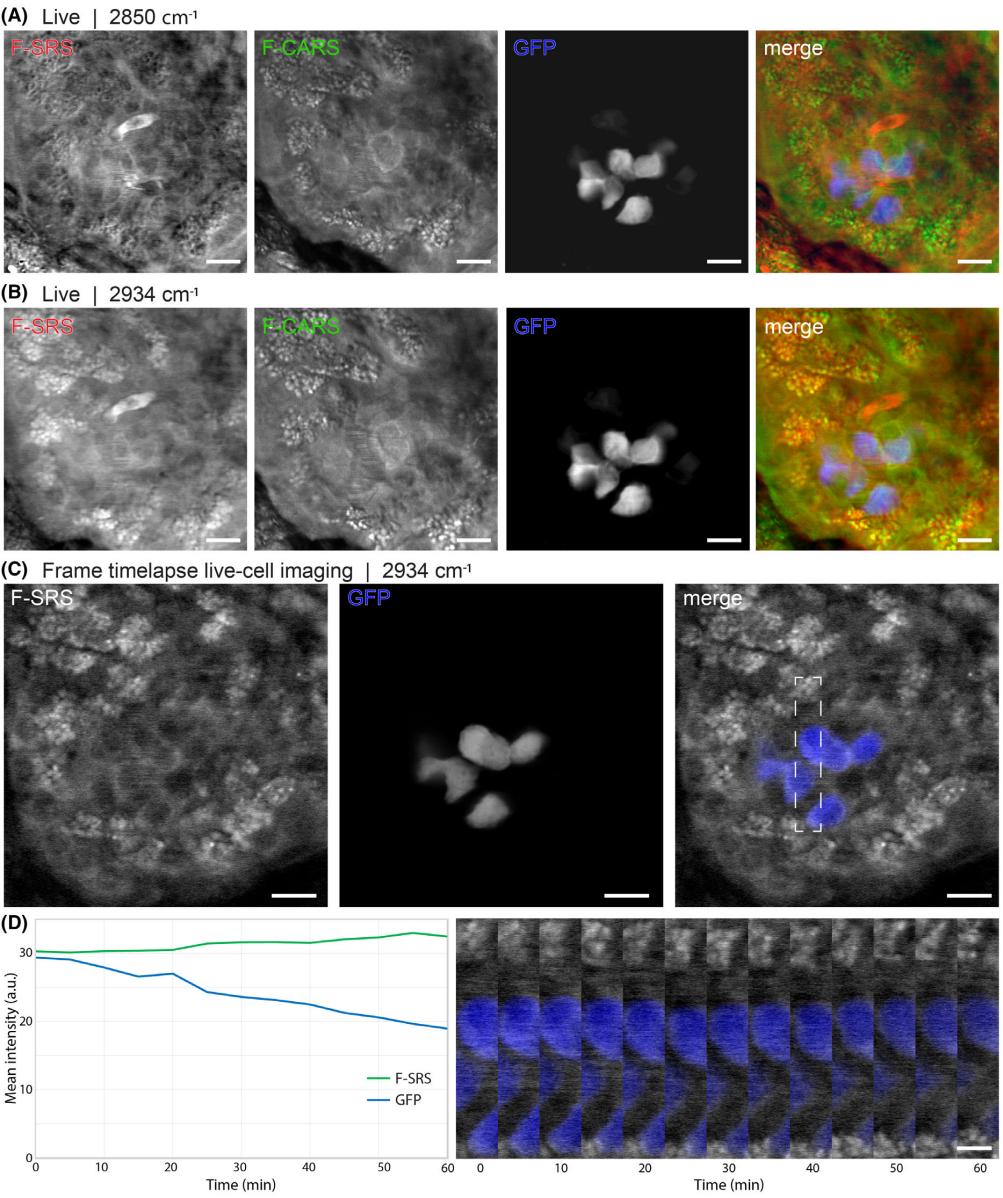
F‐SRS Raman imaging of living zebrafish does not show photobleaching, while fluorescent GFP imaging does. To show the potential of Raman for live‐cell imaging, living zebrafish were imaged over time. This also highlights the benefits of this technique, showing no photobleaching of the Raman (F‐SRS) signal compared with fluorescent imaging of GFP. (A and B) Label‐free F‐SRS (red) and F‐CARS (green) signal alongside fluorescent GFP signal (E‐SHG‐detector, blue) together with the merged channel at 2850 cm^−1^ (A) and 2934 cm^−1^ (B), respectively. (C) Snapshots taken from the time‐lapse as shown in Movie S2. Imaging for > 1 h: 13 frames were taken, every 5 min, of three focal planes (Z‐stacks). The F‐SRS (gray) and GFP signal (blue) are shown for the middle stack. (D) The mean intensity of the F‐SRS and GFP signal is plotted (left), and a kymograph of the boxed area over time is shown (right). Scale bars: 10 μm (and 5 μm for the kymograph). SRS, stimulated Raman scattering; GFP, green fluorescent protein.

18 Repeat Steps 1–3
19 Sedate the larvae using 200 mg·L^−1^ MS‐222/Tricaine
20 Embed the larvae laterally in 1% low melting agarose in 1× E3 medium with 200 mg·L^−1^ MS‐222/Tricaine in glass bottom imaging dishes. Top up with 1× E3 medium containing 200 mg·L^−1^ MS‐222/Tricaine
21 Acquire images of the larval pancreas using a Raman microscope at 28 °C. Z‐stacks were acquired with a 2 μm step size
22 Various pancreatic features and a distinct cell type are observed within the islet region (Fig. 4A,B), highlighting the presence of multiple cell types within the pancreas (Fig. S2). The entire islet region is identified in 3D using optical slices and is surrounded by exocrine granules. Moreover, while the GFP signal diminishes over time due to photobleaching, the label‐free SRS signal remains stable (Fig. 4C and Movie S2). Notably, comparable observations are detected, with endocrine cells encircled by granule‐containing exocrine cells in label‐free WT AB larvae (Fig. S3B).

The unaffected SRS signal highlights that Raman microscopy in zebrafish larvae provides stable, label‐free imaging of pancreatic structures. Thus, Raman imaging offers a promising tool for long‐term live‐cell imaging studies, which maintain the signal integrity.

### Raman application: exocrine damage leads to pancreatic chemical conversions

Expanding Raman microscopy applications from localization studies to functional analysis, facilitates label‐free detection of chemical changes in treated pancreatic cells without the need for labels. Exocrine disruption is induced by the NFP–NTR system in a zebrafish model [18].

**Fig. 5:**
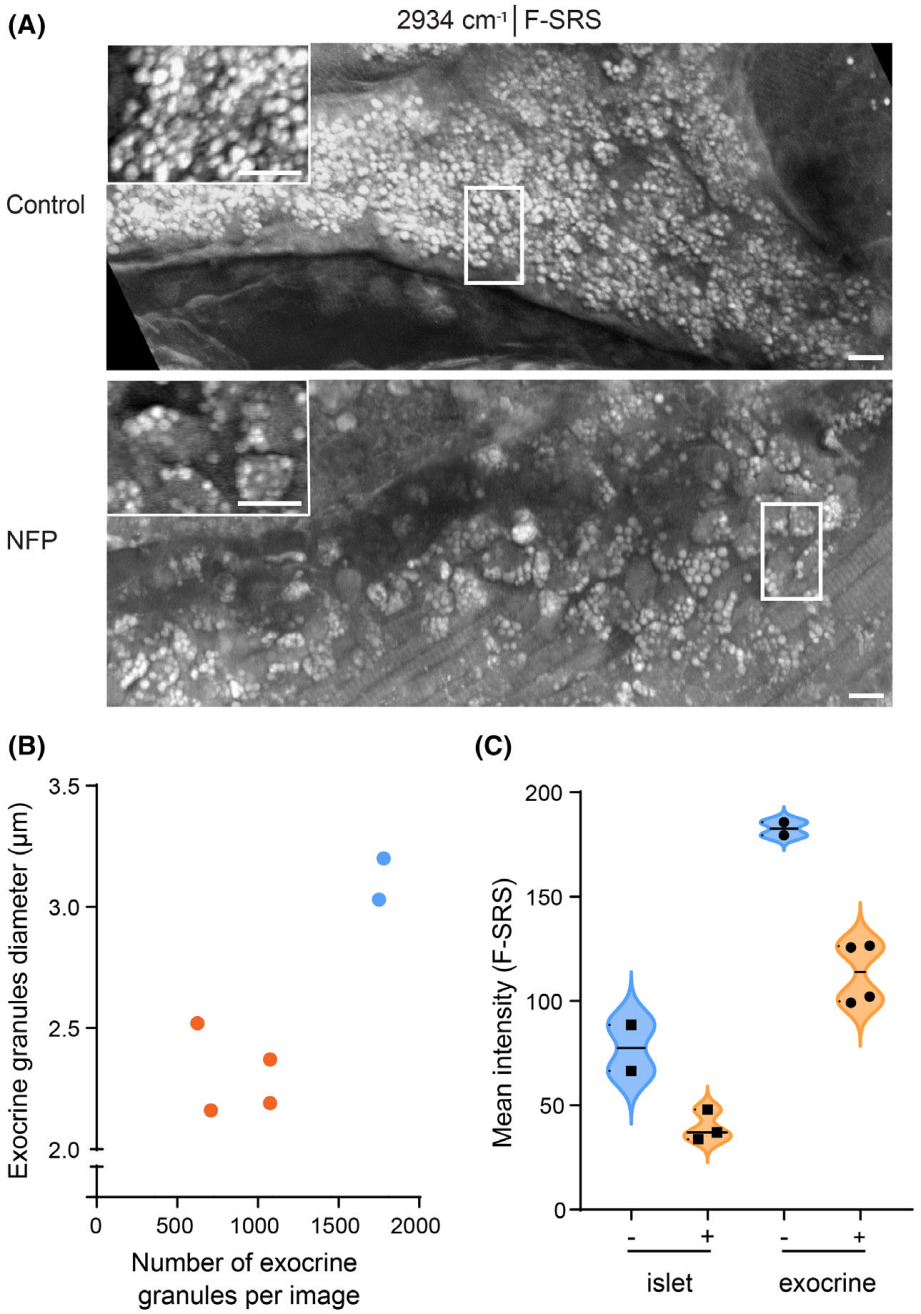
Reduced number and diameter of exocrine granules in nifurpirinol (NFP)‐treated larvae. As a proof of concept, different zebrafish were imaged that were or were not treated with NFP, a compound which induces disruption of exocrine cells. This can be seen in the Raman images, as well as in quantifications of the cell diameter and intensity of the F‐SRS signal. (A) SRS imaging of the pancreas, with zoomed‐in images of 5 μm NFP‐treated and untreated Tg(*ela3l : ntr;insulin : gfp*) at 2934 cm^−1^. Scale bars: 10 μm. (B) The relationship between the diameter of exocrine granules (in μm) and the number of exocrine granules per image in 5 μm NFP‐treated larvae (orange) versus control larvae (blue, *n* = 2–4 larvae). Control samples contain 0.1% DMSO. (C) The mean intensity of islet regions and exocrine granules at 2934 cm^−1^ after NFP treatment, indicated with a plus sign (+, in orange), compared with controls indicated with a minus sign (−, in blue; *n* = 2–3 larvae). SRS, stimulated Raman scattering.

23 Repeat Steps 1–8.
24 Acquire images of the larvae using F‐SRS and E‐CARS signal at 2850 and 2934 cm^−1^.
25 Measure the mean intensity of the F‐SRS and E‐CARS signal using the selected islet region, as well as from five randomly chosen exocrine granule regions per larva.
26 Measure the diameter of the exocrine granules (in μm). This was measured here three times for 10 randomly selected granules per sample.
27 Count the total number of exocrine granules in each image per sample.
28 Assess statistical differences between groups when applicable, using either an unpaired *T*‐test or the Mann–Whitney *U*‐test unless stated otherwise. Data are presented as violin or scatter plots. *P‐value < 0.05, **P‐value < 0.01.
29 Abnormal exocrine morphology, such as altered density of zymogenic granules, is detected through F‐SRS and E‐CARS imaging (Figs 5A and S4A,B).
30 NTR‐positive exocrine cells from zebrafish treated with NFP for 12 h show a significant reduction in both the number and diameter of exocrine granules when compared to NFP untreated (control) zebrafish (Fig. 5B).
31 Quantitative analysis of the mean intensity within the regions of interest, as outlined in [25], can provide insights into the proteins and lipids present within exocrine and endocrine compartments. A substantial decrease in the mean intensity is observed in the exocrine granules and islet region of NFP‐treated Tg(ela3l : ntr;insulin : gfp) zebrafish at 2934 cm^−1^, but not at 2850 cm^−1^.

The results suggest a potential reduction in exocrine and islet signals associated with proteins, rather than those corresponding to lipids (Figs 5C and S4C–E). Thus, Raman microscopy can reveal chemical structural transformation and disruptions in treated pancreatic cells in a label‐free manner.

## Tips & Tricks

### Sample selection, preparation, and mounting

- To avoid pigmentation and obtain good images of the larvae with a low background, add PTU on time. Since the SRS signal is only detected in the forward direction, imaged specimens should be transparent.
- The pancreas is located on the right side of 5dpf zebrafish larvae. To best position the pancreas for imaging, the relatively thick larvae are laterally embedded, as close as possible to the glass bottom of the dish. Therefore, the pancreas should be within the working distance of the objective and condenser.
- To acquire good resolution images and mount the (live) sample correctly, use low melting agarose and not normal agarose to avoid denaturation of the proteins due to heat.
- Mount the larvae in the middle of a round glass bottom dish, so the S1 condenser can reach it well.
- Make sure the microscope is calibrated and Köhlered correctly. For validation, fluorescent bead samples could be used.
- Use fresh samples (< 1 week old) for the best results

### Sample imaging

- To avoid burning or damaging your sample or detector, avoid exceeding the laser power limitation: A 300‐mW Stokes beam (1031.1 nm) and a 150‐mW tunable pump beam (720–980 nm) 2 : 1 ratio should be maintained, with a laser power of up to 50%. Since the signal is much lower in the fingerprint region (below 1800 cm^−1^), use more line accumulations and a longer pixel dwell time to properly detect the signal (in fixed samples).
- SRS is in principle background‐free, while CARS and SHG also detect autofluorescence or fluorescent signals, which become especially apparent at lower wavelengths (fingerprint region).

## Discussion

Building on Raman spectroscopy, the transition to Raman imaging allows for spatially resolved molecular insights, supported by the high sensitivity and feasibility of advanced techniques, such as SRS and CARS. Here, Raman microscopy reveals its potential for label‐free, live‐cell imaging of the region of interest, namely the pancreatic cells targeted in this study. Functional analysis of Raman data shows a significant reduction in both the number and size of zebrafish exocrine granules following induced damage, along with notable morphological changes. Additionally, disruption of the exocrine cells may lead to chemical structural changes in both exocrine and islet regions.

Although advanced fluorescence microscopy and the use of transgenic zebrafish with fluorescent labels are effective for studying various biological processes and diseases, reviewed by [26], generating transgenic models with fluorescent proteins may not always be necessary and can present challenges, such as photobleaching and the time‐consuming nature of the process. For instance, the Tg(*ela3l : ntr;insulin : gfp*) model enables the visualization of exocrine disturbances through fluorescent relocation after NFP treatment [18]. However, in the current study, the observed morphological changes in exocrine tissue as well as the decrease in the number and size of exocrine granules demonstrated the induction of exocrine damage without the need for fluorescent labeling.

Functional analysis of Raman microscopy data can provide valuable information on chemical and real‐time alterations in living organisms [27, 28]. The vibrational Raman spectrum between 450 and 3100 cm^−1^ allows detailed biomolecular analysis of the islet and exocrine cells in label‐free WT and transgenic zebrafish. Vibrational frequencies at 545, 1450, 2850, and 2934 cm^−1^ are associated with the presence of disulfide bonds, like in insulin (S‐S), aromatic rings (tryptophan), CH_2_ symmetric stretching (lipids), and CH_3_ methyl groups (proteins), respectively [21, 22, 23]. Previously, Raman spectroscopy on cryosections of human islets detected disulfide bridges and tryptophan within insulin‐ and glucagon‐producing cells, respectively [21]. While in our study, exocrine granules and the islet region are demonstrated in living zebrafish larvae, recognized using the wavenumbers 2850 and 2934 cm^−1^, respectively. However, no significant signals are detected at 545 and 1450 cm^−1^. To prevent interruptions during F‐SRS/E‐CARS scanning (450 and 3100 cm^−1^), the E‐CARS 2000 cm^−1^ filter is not replaced with a 1200 cm^−1^ filter. Given that exocrine cells have high protein production rates and 80% of the islet consists of beta cells [29, 30], the frequencies at 2850 and 2934 cm^−1^ likely correspond to the detection of proteins and lipids in the exocrine and endocrine regions. Moreover, exocrine damage induces beta‐cell stress and loss [18] and impaired beta cells cause changes in the metabolic activity [31]. Thus, the findings suggest possible modifications in the protein composition, while the lipid composition appears unchanged, in both the islet and exocrine regions following exocrine disturbance.

The thickness of 5 dpf zebrafish larvae presents challenges for focused time‐lapse imaging, making proper mounting of the larvae samples essential. To overcome this, other microscopy imaging modalities for Raman imaging can be used for thicker samples, such as light sheet Raman micro‐spectroscopy (Müller et al. [32]).

CARS signals below 1200 cm^−1^ are often too faint for reliable detection, capturing fluorescent or background signals, especially since fluorescent Tg(*ela3l : ntr;insulin : gfp*) larvae were used for pancreatic validation. While, only faint background signals were observed in E‐CARS imaging at low wavenumbers in label‐free WT larvae. Therefore, optimizing the SRS fingerprint region (lower wavenumbers, below 1800 cm^−1^) could enhance the sensitivity and depth of analysis, improving the understanding of chemical composition and structural changes.

Raman microscopy, including SRS and CARS, provides a label‐free approach to analyze cell biology, such as chemical structural transformations in pancreatic cells following exocrine damage. The ability to perform live‐cell imaging over time highlights the potential for monitoring chemical conversions in living larvae without the need for labeling. The applicability of Raman imaging extends beyond pancreatic cells, as demonstrated by the SRS imaging of zebrafish muscle, showcasing its versatility across multiple organ systems. Therefore, Raman microscopy can be used to create quantitative maps of biochemical features in human pancreatic and other organ tissues, potentially uncovering previously unrecognized abnormalities.

## Conflict of interest

The authors declare no conflict of interest.

## Author contributions

NF and EMFL designed the experiment and acquired the data. BNGG and KAS performed proof‐of‐concept pilot experiments. BNGG conceived the idea and supervised the project. NF and EMFL wrote the manuscript. All authors analyzed and interpreted the data, revised the manuscript, and provided feedback.

## Supporting information


**Fig. S1.** Raman analysis of specifically selected wavenumbers allows discrimination of various pancreatic structures in fixed, unlabeled zebrafish.
**Fig. S2.** Raman analysis of living, label‐free zebrafish allows discrimination of various pancreatic structures.
**Fig. S3.** Specific Raman signatures enable label‐free pancreatic cell characterization.
**Fig. S4.** Functional Raman analysis of the zebrafish pancreas.
**Table S1.** Acquisition time of F‐SRS, E‐CARS, SHG imaging.

**Movie S1.** Raman sweep of wavenumbers 3100 to 450 cm^−1^ reveal different features in the pancreas.
**Movie S2.** Live‐cell imaging shows a stable F‐SRS signal over time.

## Data Availability

Original microscopy images are accessible via BioImage Archive ID: S‐BIAD2128, DOI: 10.6019/S‐BIAD2128.
