# Supplementary material for: Raman‐based label‐free microscopic analysis of the pancreas in living zebrafish larvae
Source: FEBS Open Bio. 2025 Nov 25:10.1002/2211-5463.70163. Online ahead of print. doi: 10.1002/2211-5463.70163 (PMC13398842; doi:10.1002/2211-5463.70163)
Supplement: Supplementary file 1 — Fig. S1. Raman analysis of specifically selected wavenumbers allows discrimination of various pancreatic structures in fixed, unlabeled zebrafish. Fig. S2. Raman analysis of living, label‐free zebrafish allows discrimination of various pancreatic structures. Fig. S3. Specific Raman signatures enable label‐free pancreatic cell characterization. Fig. S4. Functional Raman analysis of the zebrafish pancreas. Table S1. Acquisition time of F‐SRS, E‐CARS, SHG imaging. [file FEB4-9999-0-s002.docx]

**Raman-based label-free microscopic analysis of the pancreas in living zebrafish larvae**

Noura Faraj, Eline M. F. de Lange, Klaas A Sjollema, Ben N. G. Giepmans

Department of Biomedical Sciences, University Medical Center Groningen, University of Groningen, Groningen, The Netherlands.

**Content**

- **Supplementary figures**

**Fig S1:** Raman analysis of specifically selected wavenumbers allows discrimination of various pancreatic structures in fixed, unlabeled zebrafish.

**Fig S2:** Raman analysis of living, label-free zebrafish allows discrimination of various pancreatic structures.

**Fig S3:** Specific Raman signatures enable label-free pancreatic cell characterization.

**Fig S4:** Functional Raman analysis of the zebrafish pancreas.

- **Supplementary movies**

**Movie 1:** Raman sweep of wavenumbers 3100 to 450 cm^-1^ reveal different features in the pancreas.

**Movie 2:** Live-cell imaging shows a stable F-SRS signal over time.

- **Supplementary Table**

**Table 1:** Acquisition time of F-SRS, E-CARS, SHG imaging

**Supplementary figures**


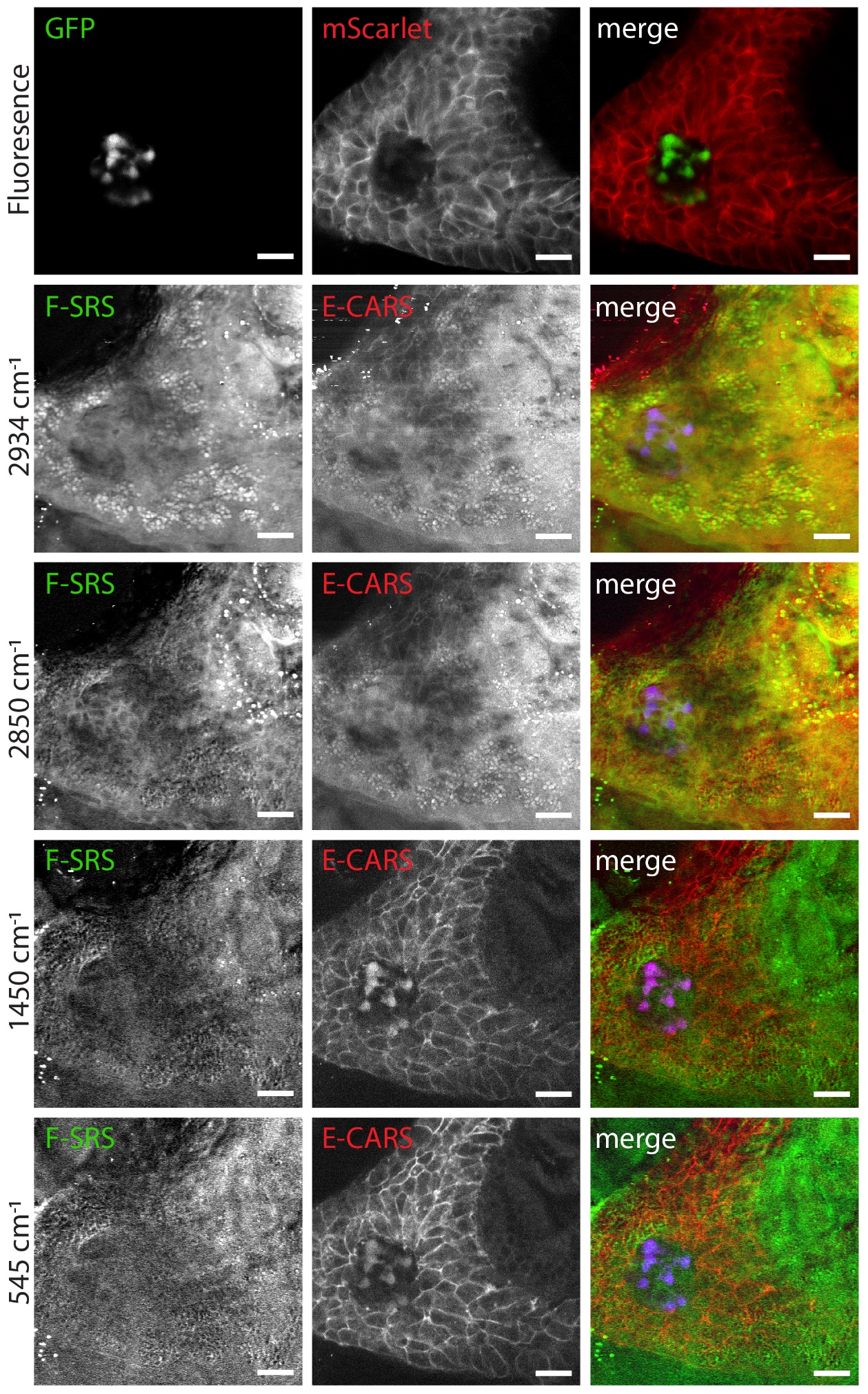


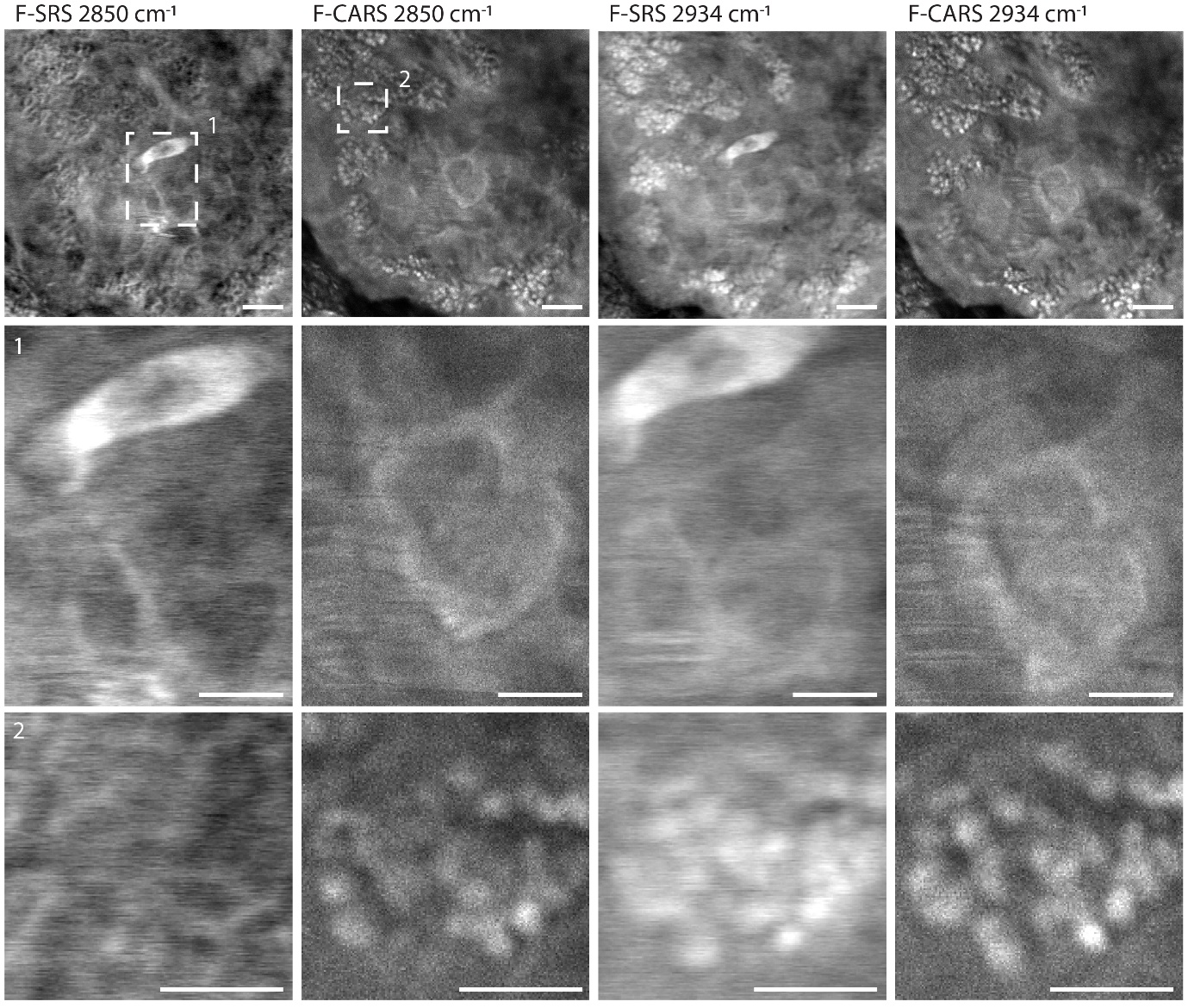
**Fig S1: Raman analysis of specifically selected wavenumbers allows discrimination of various pancreatic structures in fixed, unlabeled zebrafish.** Fluorescent images of beta cells (GFP, green) and the exocrine region of the pancreas (mScarlet, red) together with the merged channel, in fixed zebrafish (top panels). The signals are comparable to the selected peaks based on a Raman sweep of wavenumbers 3100 to 450 cm^-1^ (Fig. 3B). F-SRS signals (green), E-CARS signals (red) with fluorescent signals in lower wavenumber regions and the merged channel are shown at 2934 cm^-1^, 2850 cm^-1^, 1450 and 545 cm^-1^ respectively (bottom panels). Scale bars: 10 µm.

**Fig S2: Raman analysis of living, label-free zebrafish allows clear discrimination of various pancreatic structures.** Images of F-SRS and F-CARS at 2850 cm^-1^ (left) and 2934 cm^-1^ (right). Micrographs (Fig 4), including zoomed regions (inserts 1 and 2) from the top panel. Scale bars: 10 µm.

**
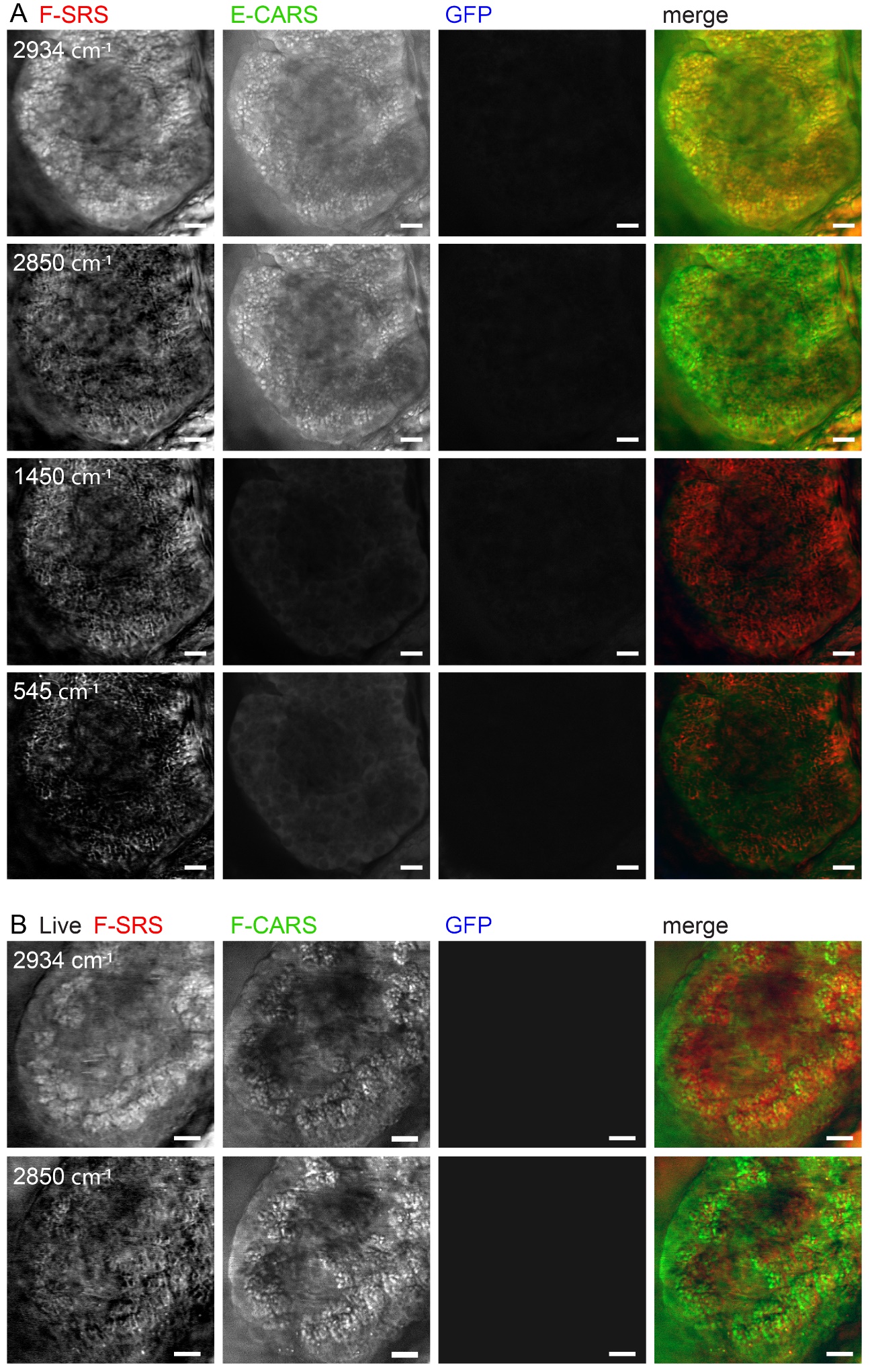

Fig S3: Specific Raman signatures enable label-free pancreatic cell characterization.**

(A): Visualization of endocrine cells and exocrine granules at selected Raman shifts (2934, 2850. 1450, and 545 cm-1) using F-SRS and E-CARS channels in 5dpf fixed, label-free wild type AB larvae, with no detectable GFP signal. (B): Imaging of living AB larvae showing F-SRS, F-CARS, and GFP signals (detected via the E-SHG detector) at Raman shifts of 2850 and 2934 cm⁻¹. Scale bars: 10 µm.


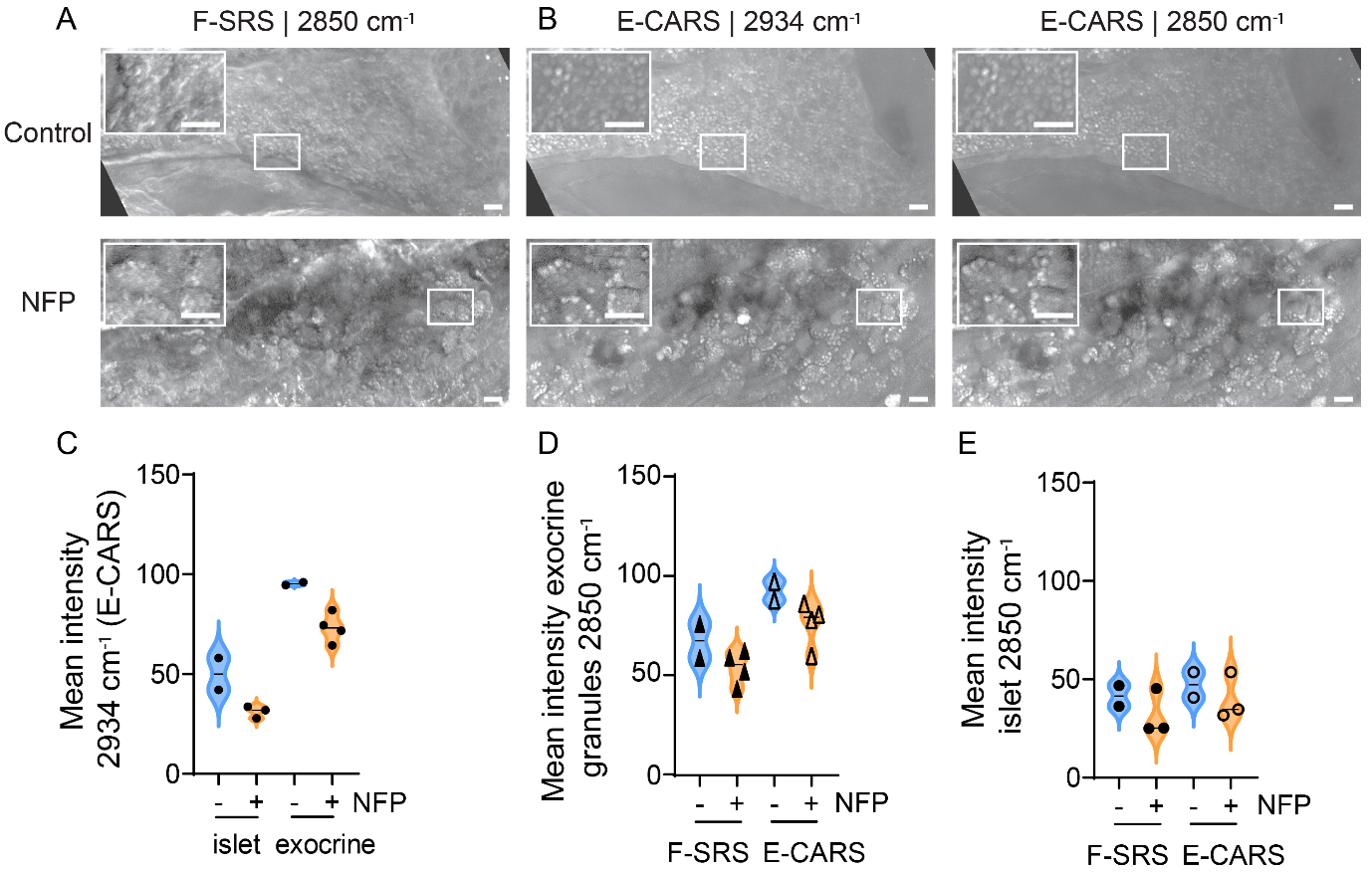


**Fig S4:** **Functional Raman analysis of the zebrafish pancreas.** (A): F-SRS imaging of the pancreas at 2850 cm^-1^ in NFP treatment vs. control. Scale bar: 10 µm. (B): F-SRS and E-CARS imaging of the pancreas at 2934, and 2850 cm^-1^ in NFP treatment vs. control. Scale bars: 10 µm. (C-E): E-CARS mean intensity of the islet and exocrine granules at 2934 cm^-1^, and F-SRS and E-CARS mean intensity of the islet and exocrine granules at 2850 cm^-1^ (n=2-4 larvae). Control is 0.1% DMSO, indicated with a minus sign (-, in blue) compared to NFP treatment indicated with a plus sign (+, in orange).

**Supplementary movies**

**Movie 1: Raman sweep of wavenumbers 3100 to 450 cm^-1^ reveals different features in the pancreas.** Scan of F-SRS signal from 3100 to 450 cm^-1^ shown with a step size of 1 nm each. (A): F-SRS signal, (B): corresponding E-CARS signal. The wavenumber is indicated in the top left corner.

**Movie 2: Live-cell imaging shows a stable F-SRS signal over time.** F-SRS signal (gray) and GFP signal (blue) shown over time, at 2934 cm^-1^. A picture is taken every 5 min, and continued for 60 minutes. The time is indicated in the top left corner.

**Supplementary Table**

Table 1: Acquisition time of F-SRS, E-CARS, SHG imaging

| **Image procedure** | **Acquisition time** |
| --- | --- |
| Fluorescent image | 20 seconds |
| Raman image | 20 seconds * |
| Z-stack of 18 slices | 4 minutes |
| λ-scan of 205 images | 1 hour |
| Tuning | ~ 1 – 2 minutes |

* The range of Raman acquisition time is between 10 and 40 seconds. Images at high wave numbers take 10 seconds. While at lower wave number or live imaging, images take 40 seconds to acquire.
