# Supplementary material for: Raman‐based label‐free microscopic analysis of the pancreas in living zebrafish larvae
Source: FEBS Open Bio. 2025 Nov 25:10.1002/2211-5463.70163. Online ahead of print. doi: 10.1002/2211-5463.70163 (PMC13398842; doi:10.1002/2211-5463.70163)
Supplement: Supplementary file 2 — Movie S1. Raman sweep of wavenumbers 3100 to 450 cm−1 reveal different features in the pancreas. Movie S2. Live‐cell imaging shows a stable F‐SRS signal over time. [file FEB4-9999-0-s001.zip › Movies.docx]

Dear Editor,

The full manuscript including figuresd and movies exceeds the upload limit. Please find downloadlinks for the movies below:

<http://www.nanotomy.org/OA/Faraj2026FEBSopenbio/Faraj_Movie1.avi>

<http://www.nanotomy.org/OA/Faraj2026FEBSopenbio/Faraj_Movie2.avi>

Thanks, Ben Giepmans
